# Distal radius fractures with diaphyseal involvement: fixation with fixed angle volar plate

**DOI:** 10.1007/s10195-011-0147-x

**Published:** 2011-07-07

**Authors:** Michele Rampoldi, Dante Palombi, Donatella Tagliente

**Affiliations:** 1 Centro Traumatologico Ortopedico—Roma—ASL RMC, U.O.C. Chirurgia della Mano, Microchirurgia e Reimpianto Arti, Rome, Italy; 2 Policlinico Tor Vergata UOC Ortopedia e Traumatologia A, Rome, Italy

**Keywords:** Distal radius fractures, Diaphyseal involvement, Internal fixation, Fixed angle volar plate

## Abstract

**Background:**

The purpose of this study was to evaluate the outcome of fractures of the distal radius with metaphyseal and diaphyseal involvement treated with fixed angle volar plates.

**Materials and methods:**

Twenty-one patients with fracture of the radius involving the diaphyseal, metaphyseal and epiphyseal parts were treated with fixed angle plate fixation through an extended volar Henry’s approach. Circle wire loops, screws and intrafocal wire fixations were associated in 12 cases. Coexisting ulnar fractures were fixed with plates or K-wires in 8 cases. All patients were prospectively followed using radiographs, physical examination, and DASH (Disabilities of the Arm, Shoulder and Hand) scores.

**Results:**

All fractures except one, which needed a secondary bone graft to achieve consolidation, united by an average of 90 days. One case developed a radioulnar synostosis. Radiographs showed optimal reduction in 17 of 21 cases, with restoration of radial length in all cases and a neutral average ulnar variance. Nonanatomical reduction was associated with the worst results (*P* = 0.0006). Flexion and extension averaged 62.8° and 73.8°, and pronation and supination 85.2° and 80.2°, respectively. The average DASH scores were 30 points at 3 months, 14 points at 6 months, and 6.7 points at the time of final follow-up (at an average of 11 months). According to the Mayo wrist rating system, 14 patients showed excellent results, 5 showed good results, and 2 showed fair results.

**Conclusion:**

Fixed angle volar plates were demonstrated to be a safe and efficient treatment in these challenging fractures.

## Introduction

Fractures of the distal radius involving the metaphyseal and diaphyseal junction are commonly the result of high-energy trauma, and represent a challenge for the orthopedic surgeon. Fractures are often comminuted: optimal reduction, restoration of normal radial length, and a correct radioulnar relationship may be difficult to achieve. Only a few reports in the literature have studied these lesions, and the best treatment approach is still the source of debate.

Treatment options include external fixation, distraction plate fixation using a dorsal approach, standard 3.5 mm LCP plates, and fixed angle volar plates.

Volar plate fixation has been recognized to be an effective and safe treatment in unstable distal radius fractures [1, 2]. Long volar plates are available in most surgical instrumentation boxes to manage distal radial fractures that extend to the diaphysis. The reasoning behind this treatment is that long volar plates can reduce the distal radius, stabilize the metadiaphyseal junction, and fix the diaphysis firmly, restoring the articular congruity and relationship, as well as the radial length and alignment. Wrist immobilization is limited to 3 or 4 weeks, allowing early functional recovery.

We report the results of 21 cases treated with this technique using an extended Henry’s volar approach.

## Materials and methods

Twenty-one fractures of the distal radius that extended to the diaphysis were prospectively studied (see Table 1).

Patients included in this study had a recent, untreated, closed distal radial fracture extending from the proximal up to the middle third of the diaphisys. Patients with open fractures, those receiving delayed treatment (>20 days from the trauma), or those previously treated with other systems were excluded from the study. All fractures were treated with a long fixed angle volar plate.

The study included 6 female and 15 male patients. The average age of the patients was 41 (range 24–73 years).

Most of the fractures were due to car or motor vehicle accidents (12 cases); domestic accidents were responsible in 5 cases, work injuries in 3, winter sports in 1. Eight cases had a concomitant distal ulnar fracture, while 6 cases were associated with other injuries—lower limb fractures or ligament sprains (4 cases), homolateral coronoid fracture (1 case) and contralateral radial capitellum fracture (1 case). According to the AO comprehensive classification, fractures have been classified as A3.3 (12 cases) and C2–C3.3 (9 cases). All patients had preoperative standard radiographs; CT scan was executed in 6 cases with major articular involvement.

Operations were performed 1–5 days from the trauma (mean delay 2.6 days). Patients were operated on under locoregional anesthesia using a tourniquet.

The fracture was exposed via a standard Henry’s access extended proximally to the diaphysis. Articular involvement, when present, was accurately reduced; restoration of length and correct alignment was verified with an image intensifier, and the fracture was fixed with the plate. Three different fixed angle long volar plates were used: DVR (Hand Innovation LLC, Miami, FL, USA) plates in 11 cases, Acu Lock (Acumed^®^) in 4, and LCP Synthes plates (Synthes Ltd.) in the remaining 6 cases.

**Fig. 1:**
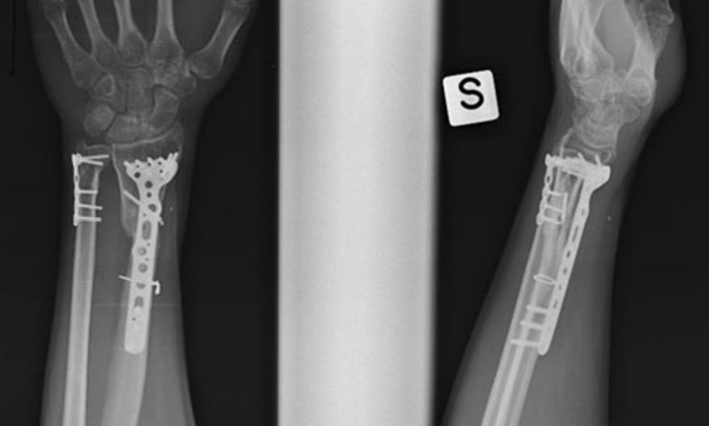
X-rays at 6 months of an A3 fracture. Plate fixation (Acu-lock, Acumed^®^) was associated with circle wire loop and single screw fixation; the ulnar fracture was plated

**Fig. 2:**
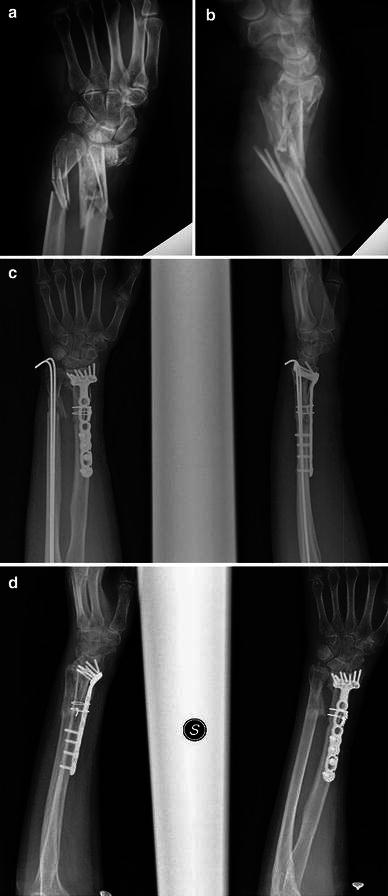
**a**, **b** C3 fracture in a 73-year-old female. **c** Plate fixation (DVR, Hand Innovation LLC, Miami, FL, USA) of the radius and K-wires fixation of the ulna. **d** Bone healing at 3 months

Circle wire loops and screw-specific fragment fixation were associated with radial plating in 12 patients (Fig. 1); no fractures were bone grafted. Distal ulnar fractures were fixed with small fragment plates (Fig. 1) in 4 cases and with K-wires (Fig. 2) in the other 4. Percutaneus pin fixation was used in cases of stable retention of the ulnar head after closed reduction, while ORIF was used in patients with unstable fractures of the distal ulna.

**Table 1:** Case series

Patients	FR. type	Plate	Ulnar fract.	Reduction	Union	Complication	Dash	Mayo wrist score	Flexion	Extension	Pronation	Supination
SC	A3.3	DVR		+	+	−	2	Excell	65	80	90	90
BA	A3.3	ACULOK	+ Plate	+	+	−	4	Excell	70	80	85	85
DD	A3.3	LCP		+	+	−	4	Good	65	70	90	80
QG	A3.3	LCP	+ K-wires	+	+	−	7	Good	65	75	85	85
CF	A3.3	DVR		+	+	−	7	Excell	70	80	90	90
CA	A3.3	LCP		−	+	R-U synosthosis	18	Poor	45	45	80	70
SP	A3.3	LCP		+	+	−	4	Good	50	75	90	75
CF	A3.3	DVR	+ K-wires	+	− Bone graft	Nonunion	7	Good	70	75	90	80
TA	A3.3	DVR		**−** RUD incongr.	+	−	11	Satisf.	65	75	70	65
CMD	A3.3	DVR		+	+	−	2	Excell	75	90	90	90
GD	A3.3	LCP	+ Plate	+	+	−	4	Good	60	70	85	85
AV	A3.3	LCP		+	+	−	4	Excell	65	75	90	80
GC	C2.3	DVR	−	+	+	−	4	Excell	65	75	90	90
FR	C2.3	DVR	+ Plate	+	+	−	2	Excell	70	80	90	80
PA	C2.3	ACULOK		− Art. step	+	−	16	Poor	40	60	80	70
DS	C2.3	ACULOK		+	+	−	9	Satisf.	60	70	75	75
FN	C3.3	DVR	+ K-wire	+	+	−	7	Good	65	75	85	80
FF	C3.3	DVR	+ Plate	−	+	−	9	Satisf.	60	70	80	75
GB	C3.3	ACULOK		+	+	−	7	Good	65	80	85	80
NL	C3.3	DVR	+ K-wires	− Art. step	+	−	11	Satisf.	60	70	80	70
DM	C3.3	DVR		+	+	−	3	Excell	70	80	90	90

### Postoperative care

Eleven patients had a rigid splint keeping the wrist in slight extension, with the elbow free, for 3 weeks. After this period they started gentle active motion of the wrist, avoiding weights and heavy manual activities; carefulness regarding torsion movements was recommended.

Ten patients with multi-fragmented and more unstable fractures were immobilized with a below-elbow cast for 4 weeks that was replaced with a removable splint for a minimum of two further weeks. Rehabilitation was started and carried out the same way as for the other patients.

Patients were evaluated monthly with standard X-rays and clinical examinations according to the Mayo wrist score system; DASH scores was assessed at 3, 6 and 12 months from the operation.

Results were statistically analyzed using the Kruskal–Wallis test. The outcome was compared to the type of fracture (extrarticular A fractures and articular C fractures), the presence or absence of an associated ulnar fracture, and the quality of reduction. Radiographical parameters of good reduction (+) were: radial inclination 20°(±5), volar tilt 10°(±5°), radial length 10 mm, neutral ulnar variance, no articular step-off. Any differences in these parameters were classified as imperfect reduction (−). The mean duration of follow-up was 13 months (range 9–18 months).

Passive ROM of the wrist (flexion, extension, radial and ulnar deviation) and active forearm pronation and supination were evaluated using a double-armed goniometer. A Jamar dynamometer was used to test grip strength. Data were recorded while the upper extremity was kept in a standardized position (patient seated with the shoulder adducted and neutrally rotated, elbow flexed at 90°, forearm and wrist in neutral position); values were compared to those in the opposite hand. Pain was evaluated through a verbal scale (no pain—mild pain—severe pain).

## Results

**Fig. 3:**
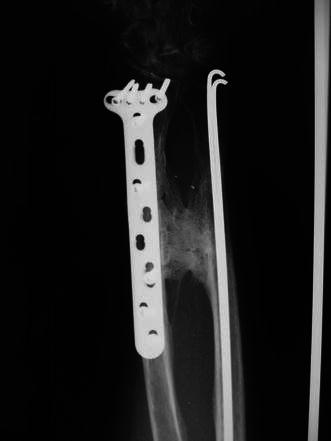
Radioulnar synostosis at 9 months after osteosynthesis (Synthes, Ltd.) of an A3 fracture

Twenty out of 21 fractures united in an average of 90 days. One fracture, at 4 months from operation, showed incomplete healing of the diaphyseal fracture and was consequently bone grafted; consolidation was achieved 2 months later. One patient developed a radioulnar synostosis (Fig. 3) and was reoperated on 9 months after the former surgery, leading to almost complete recovery of the forearm rotation.

Radiographs showed recovery of normal radial anatomy—radial inclination 20°, volar tilt 10°, radial length 10 mm, neutral ulnar variance, no articular step-off—in 10 of 12 A3 fractures. Among the other 2 cases, there was a case associated with ulnar head fracture, which healed with mild ulnar dorsal subluxation and articular incongruity, clinically associated with incomplete supination; the other case showed a neutral volar tilt. Of the 9 fractures with articular involvement (C2–C3), 7 obtained optimal reduction while 2 cases showed mild articular incongruity due to radial articular steps (1 mm). Radial length was restored in all cases; ulnar variance averaged 0 mm.

At the final follow-up, flexion and extension averaged 62.85° [range 40–80°, 95% confidence interval (CI) ±3.92, standard deviation (SD) 8.5982] and 73.85° (range 45–90°, CI ±4.07, SD 8.931); pronation and supination 85.24° (range 70–90°, CI ±2.64, SD 5.80) and 80.23° (range 65–90°, CI ±3.49, SD 7.661), respectively.

**Fig. 4:**
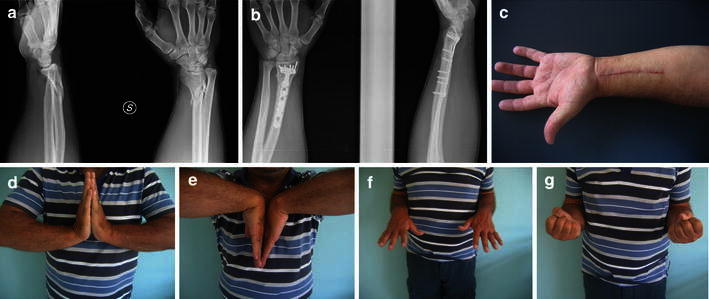
Excellent radiographical (**a**, **b**) and clinical (**c**, **d**, **e**, **f**, **g**) results at 6 months after plating (DVR Hand Innovation LLC, Miami, FL, USA) a C2 fracture in a 65 year-old male

The average DASH scores were 30 points at 3 months, 14 points at 6 months and 6.76 points (range 2–18, CI ±2.003, SD 4.39) at the time of final follow-up (at an average of 11 months). According to the Mayo wrist rating system, 4 patients had excellent results (90–100), 10 had good results (80–90) and 7 had satisfactory results (60–80). A3 fractures showed 9 good or excellent results and 3 satisfactory results. C2 and C3 fractures revealed 5 excellent (Fig. 4) or good results and four satisfactory results.

The type of fracture (type A 12 cases, mean DASH rank 10; type C 9 cases, mean DASH rank 12.3) was not significantly correlated with the outcome (*P* = 0.2061). An associated ulnar fracture (8 cases, mean DASH rank 11.4) produced no statistical differences in outcome (*P* = 0.4286) with respect to patients with an isolated radial fracture (13 cases, mean DASH rank 10.8). The only factor that was found to be correlated with the results was the quality of the reduction. Patients who healed with imperfect reduction (5 cases, mean DASH rank 18.9) had worst results than those whose fractures were satisfactorily reduced (16 cases, mean DASH rank 8.5) (*P* = 0.0006).

We report 2 major complications, a delay of healing and a radioulnar synostosis, that underwent secondary surgery. Final results were both graded as satisfactory; almost complete recovery of forearm rotation was achieved.

## Discussion

High-energy trauma of the distal forearm may result in fractures that simultaneously involve the diaphysis and the metaepiphyseal parts of the bones; soft tissue damage may be associated. These lesions represent a major treatment challenge, as the radial length, articular congruity, and the correct relationship of the distal radioulnar joint may be difficult to restore. External fixation respects the biology of the consolidation process and is mainly indicated in multifragmented and comminuted fractures. This technique may be effective at restoring radial length but it often fails to correct palmar angulations; moreover, indirect reduction through ligamentotaxis may not effectively reduce articular fragments in fractures with major joint involvement. Excessive distraction of the hardware, to obtain satisfactory reduction, can result in delayed union, nonunion, complex regional pain syndrome (CRPS) or digital stiffness [3, 4]. Despite the fact that randomized trials do not provide strong evidence regarding the type of surgical intervention that is the most appropriate treatment for fractures of the distal radius in adults, superior functional and radiological results of ORIF with respect to external fixation have been reported recently [5]. Standard straight plates result in optimal diaphyseal stabilization but may be inadequate for fixing metaphyseal and epiphyseal fractures. When positioned on the volar aspect of the radius, straight plates are too bulky and may create attritional damage to flexor tendons; they need to be bent to follow the radial volar inclination, and are not sufficiently wide to adequately fix the epiphysis and capture small articular pieces for such fractures. A new technique described by Ginn et al. [6] involves “bridging” the fracture using a standard 3.5 mm plate applied dorsally and fixed in distraction from the radius to the third metacarpal shaft distally. The distraction plate technique also uses the concept of ligamentotaxis and, like external fixation, is especially indicated for intra-articular fractures with small, comminuted fragments which may be difficult to manage with plates or other nonbridging methods; when distraction fails to obtain adequate reduction, the use of bone grafts, K-wires and supplementary screws are included in the procedure. The hardware is removed after radiographic evidence of consolidation (mean time: 124 days) and wrist motion has been initiated. Excellent clinical and radiographical results are reported with this technique [7]. Disadvantages of this method include the long period of immobilization of the wrist, the need for a second operation to remove the hardware, potential attritional damage to the extensor tendons, difficulty in reducing and stabilizing the volar fragments of the distal radius, and the risks related to overdistraction of the radiocarpal joint (CPRS, finger functional impairement) [3, 4].

Fixed-angle volar plates can adequately stabilize articular fragments and both volar and dorsal comminution, even in osteoporotic bone [8, 9]. Optimal positioning of the plate, with screws and pins placed in the subchondral position, restores radial length and radial and palmar tilt, acting as an internal fixator; indirect reduction of the dorsal aspect of the radius avoids damaging bone vascularity in comminuted fractures, reducing or avoiding the need for bone grafting. Using long plates, the metaphyseal and distal diaphyseal fracture is bypassed and the plate fixed proximally with at least three screws in the healthy diaphyseal bone. In multifragmented and highly comminuted fractures, multiple wire loops or single cortical screws can be used to improve stability and the reduction of the metadiaphyseal part of the radius. The rationale behind this technique is to obtain optimal stability of the fracture, restoring radial length and correct radioulnar and radiocarpal relationships, avoiding interference with soft tissues, and allowing an early functional recovery. As the plate is positioned deep and covered by muscles, interference with tendons is rare; elsewhere, there is no need to remove the hardware. Associated ulnar head fractures are treated with percutaneous pin fixation or ORIF depending on the stability of the fracture [10, 11]. In our cases, this technique led to the consolidation of all fractures except one that needed a secondary bone grafting. Optimal reduction was achieved in most cases (16 out of 21); in particular, restoration of radial length was obtained in all cases with average ulnar variance of 0°. Two patients healed with an articular step; clinical results were graded as good and fair. No case showed loss of reduction during the treatment. Imperfect reduction was found to be associated with higher DASH scores (*P* = 0.0006). A clinical evaluation revealed satisfactory results in 19 cases out of 21. Two cases had poor results—one associated with articular incongruity and one due to radioulnar synosthosis. The latter case was operated on 12 months from the first intervention with almost complete recovery of forearm rotation. Extra-articular A3 fractures showed better results than articular C2 and C3 fractures, but no significant differences were noted among these two groups (*P* = 0.2). Associated ulnar fractures are generally the consequence of a high-energy trauma; despite that, they did not produce significant effects on the outcome (*P* = 0.4). Even though the fractures appeared extremely comminuted in some cases, bone grafts or substitutes were never used. In C3 fractures with multiple small articular fragments, adequate fixation with periarticular plates may be difficult and, in these cases, some authors advocate the association of an external fixator to avoid articular surface collapse [12, 13]; however, in this series of cases, external fixation was never used to support the plate, and we only noted two minor articular steps of the radius.

Very few reports in the literature have studied the treatment of these complex fractures. The sample size of this study is not very large, but it is similar to previously reported series. Despite some points of weakness (inhomogeneous samples with respect to the type of fracture, age and plates used), our study seems to demonstrate the effectiveness of volar long plating for treating distal radius fractures that extend proximally to the radial diaphysis.
